# Human papilloma virus correlates of high grade cervical dysplasia in HIV-infected women in Mombasa, Kenya: a cross-sectional analysis

**DOI:** 10.1186/s12985-018-0961-3

**Published:** 2018-03-27

**Authors:** Sonia Menon, Stanley Luchters, Rodolfo Rossi, Steven Callens, Mandaliya Kishor, Johannes Bogers, Davy vanden Broeck

**Affiliations:** 1 International Centre for Reproductive Health (ICRH), Department of Obstetrics and Gynaecology, Ghent University, De Pintelaan 185 P3, 9000 Ghent, Belgium; 2 Burnet Institute, Melbourne, VIC Australia; 3 Department of Epidemiology and Preventive Medicine, School of Public Health and Preventive Medicine, Monash University, Melbourne, VIC Australia; 4 Primary Health Care Services, International Committee of the Red Cross (ICRC), Geneva, Switzerland; 5 Department of Internal Medicine & Infectious diseases, University Hospital, Ghent, Belgium; 6 ICRH, P.O.Box 91109, Mombasa, 80103 Kenya; 7 Faculty of Medicine and Health Sciences, AMBIOR (Applied Molecular Biology Research Group), Laboratory of Cell Biology & Histology, University of Antwerp, Melbourne, Belgium

**Keywords:** Human papilloma virus, Potentially high risk/high-risk HPV genotypes, HPV viral load, Co-infections, Pairings, CIN 2 +, Kenya

## Abstract

**Background:**

Women living with HIV are at increased risk to be co-infected with HPV, persistent high-risk (HR) human papillomavirus (HPV) infection and increased HR HPV viral load, which make them more at risk for cervical cancer. Despite their inherent vulnerability, there is a scarcity of data on potential high risk (pHR) and HR HPV genotypes in HIV- infected women with cervical dysplasia and HPV-type specific viral load in this population in Sub Saharan Africa.

The aim of this analysis of HIV-infected women was to explore the virological correlates of high-grade cervical dysplasia (CIN 2+) in HIV-infected women, thereby profiling HPV genotypes.

**Method:**

This analysis assesses baseline data obtained from a cohort study of 74 HIV-infected women with abnormal cytology attending a Comprehensive Care Centre for patients with HIV infection in Mombasa, Kenya. Quantitative real-time PCR was used for HPV typing and viral load.

**Results:**

CIN 2 was observed in 16% (12/74) of women, CIN 3 in 23% (17/74), and, invasive cervical carcinoma (ICC) in 1% (1/74) of women. In women with CIN 3+, HPV 16 (44%), HPV 56 (33%), HPV 33 and 53 (HPV 53 (28%) were the most prevalent genotypes. HPV 53 was observed as a stand-alone HPV in one woman with ICC.

A multivariate logistic regression adjusting for age, CD4 count and HPV co-infections suggested the presence of HPV 31 as a predictor of CIN 2+ (adjusted odds ratio [aOR]:4.9; *p* = 0.05; 95% (Confidence Interval) [CI]:1.03–22.5). Women with CIN2+ had a significantly higher viral log mean of HPV 16, (11.2 copies/ 10,000 cells; 95% CI: 9.0–13.4) than with CIN 1.

**Conclusion:**

The high prevalence of HPV 53 in CIN 3 and as a stand-alone genotype in the patient with invasive cervical cancer warrants that its clinical significance be further revisited among HIV-infected women. HPV 31, along with elevated means of HPV 16 viral load were predictors of CIN 2 + .

## Background

Kenya is home to the world’s fourth-largest HIV epidemic in the world. In 2013, an estimated 1.6 million people were living with HIV and roughly 57,000 people died from AIDS-related illnesses [1]. Cervical carcinoma, an AIDS-related cancer, is the most common female cancer in sub-Saharan Africa; [2] it has become the second most prevalent cancer among women in Kenya, after breast cancer, and its incidence is increasing [3].

Distinct precancerous stages or pre-invasive precursor lesions called cervical intraepithelial neoplasia (CIN), or dysplasia can be discriminated before becoming invasive cervical cancer (ICC). CIN can be histologically graded into mild dysplasia (CIN 1), moderate dysplasia (CIN 2), and severe dysplasia to carcinoma in situ (CIN 3) [4].

Human Papilloma Viruses (HPV), a sexually transmitted DNA virus are double-stranded DNA viruses, considered the primary etiological agents in cervical intraepithelial neoplasia and cancers. “High-risk”, (HR) include HPV 16, 18, 31, 33, 35, 39, 45, 51, 52, 56, 58, 59, 68 and due to lack of evidence of biological activity in tumour tissues, HPV26, 53, 66, 67, 70, 73, and 82 are classified as probably or possibly high risk [5].

It is well recognized that among the 15 HR HPV genotypes, HPV 16 and HPV 18 confer the greatest risk for CIN 2 or worse because these two genotypes are associated with approximately two thirds of all invasive cervical carcinomas [6]. Several countries, including Kenya, have licensed and adopted the bivalent HPV vaccine (Cervarix™) that protects against HPV genotypes 16 and 18 and the quadrivalent vaccine (Gardasil™) that protects against HPV genotypes 6, 11, 16 and 18 [7]. In 2014, a nonavalent vaccine, not yet commercialized in Kenya, containing the most frequently detected types in ICC worldwide, HPV types 31, 33, 45, 52, and 58 antigens, [8] will have direct implications for cervical cancer incidence and prevention in all regions of the world with the potential to prevent almost 90% of ICC cases worldwide.

If viral persistence is established, a variety of host cofactors may act upon the immune system and the tissue microenvironment in the cervix to induce development of cervical lesions. [9] A relationship has been established between HIV immunosuppression and multiple HPV infection, [10, 11] which has been attributed to the inability to clear HPV infections and to reactivate latent HPV infections, [10, 12–14]. Moreover, certain viral risk factors may also play a role in establishing viral persistence. It has been suggested that high HPV viral load may be aetiologically associated with cervical disease pathogenesis, although studies have yielded conflicting results. [15–20].

Moreover, epidemiological knowledge of potential high-risk (pHR) HPV types is limited, mainly because commercial molecular assays focus on HR HPV genotypes. Data on pHR genotypes in HIV-infected women are even scarcer, although it can be hypothesized that they might play a role in HPV related diseases in HIV positive women [21, 22]. This can be attributed to the fact that HIV infected women harbor a higher prevalence and broader range of HR HPV, and HPV 16 does not figure as prominently in HIV positive women. [23, 24].

Our analysis purported to test our two a priori hypotheses: first that single pHR and HR HPV genotypes in HIV-infected women are not independent predictors of factors of CIN 2+, but involve synergistic mechanisms, and second that HPV 16 viral load may be correlated with CIN 2+. The objectives of this secondary analysis were to determine the most prevalent genotype-specific distribution of HPV among women with CIN 2+, and to assess whether specific pHR and HR HPV genotypes and their respective viral load are associated with CIN 2 + .

## Methods

To examine the epidemiology of type-specific HPV infections, we carried out a cross-sectional analysis of all 74 HIV-infected women. This cross-sectional analysis based on primary data collection and record reviews adhered to the methodological guidelines recommended in the STROBE document on observational studies [25].

Between November 2005 and April 2006, women attending the Comprehensive HIV Care Centre (CCC) at Coast Provincial General Hospital in Mombasa, Kenya were informed about the study and were offered on site cervical cancer screening with conventional Pap smear, in addition to a general medical examination and routine blood tests, including CD4 cell count. Women were enrolled if they were HIV positive and diagnosed with squamous intra-epithelial lesions (SIL) by Pap smear, were between 20 and 50 years of age, not pregnant and did not have a history of hysterectomy or cervical cancer. Cervical sampling for HPV testing was done. During the enrolment visit, socio-demographic data were collected.

Written informed consent was obtained from all participants. Illiterate women elected a person who signed on their behalf after thorough explanation. Ethical approval for the study was given by the Ethics Committee of Ghent University Hospital and from the Ethics and Research Committee of the Kenyatta National Hospital (Ref number: Ref: KNH-ERC/01/3618). Six hundred HIV infected women were tested to reach a cohort of 74 HIV women with abnormal cytology.

### Biologic specimens

Blood plasma samples were taken and a gynaecological examination was done with speculum insertion, prior to collection of endocervical and high vaginal swabs. Cervical samples were collected using a cervix brush (Cervex-brush®, Rovers®, Oss, The Netherlands), and cervical cytology was assessed with conventional Papanicolaou (Pap) smears. Histology of the biopsy specimens were processed and read by a qualified histopathologist. An external cytopathologist provided quality control by reviewing all cases. A diagnosis to each case was assigned according to the Bethesda 2001 criteria [26]. The cervix brush tips were preserved in a liquid-based cytology collection medium (SurePath®, Tripath Imaging Inc., Burlington, North Carolina, USA) and stored at 4 °C and shipped to Belgium for HPV testing.

### Sample

Samples were collected according to the method described by Micalessi et al. 2012 [27]. Cervical cells were collected into an ethanol-based preservative (Surepath TM, Tripath Imaging, Burlington, NC, USA) using the Cervex-Brush ® or Cervex-Brush ® Combi (Rovers Medical Devices B.V., KV Oss, The Netherlands), and were processed into thin-layer LBC preparations using the fully robotic Autocyte PREP system (Tripath Imaging, Burlington, NC, USA) [28]. Upon finalizing the LBC preparations, 800 μL of the remaining cell suspension was used for DNA extraction.

### HPV DNA extraction, detection and typing

HPV testing was done as described by Depuydt et al. (2006) in an accredited laboratory (ISO certification: ISO15189)[29]. Briefly, HPV DNA was extracted from exfoliated cervical cells using the standard proteinase K-based digestion protocol. Cells were incubated with proteinase K solution (100 μg/ml) for three hours at 55 °C. DNA was then further purified by spin column chromatography. HPV types were determined using a series of real-time PCR reactions with specific primers and TaqMan® (Invitrogen, La Jolla, USA) probes for HR- HPV types 16, 18, 31, 33, 35, 39, 45, 51, 52, 56, 58, 59, 68, [30] including the pHR HPV genotypes 53 and 66**.** Low-risk HPV types 6, and 67 were also detected. HPV viral load was detected by the use of HPV type-specific real-time TaqMan PCR assays.

Assays were normalized to a reference gene. A calibrator was included in every run and a standard curve was used to convert the signal to viral load. Detection limits were described by Micalessi et al. (2012) for each primer or probe set [27].

A standard national testing algorithm was used for HIV diagnosis using rapid immunoassays: Uni-Gold™ Recombigen® HIV (Trinity Biotech plc, Bray, Ireland) and Determine® HIV-1/2 (Abbott Japan co Ltd, Minato-Ku, Tokyo, Japan). In the case of indeterminate results, an enzyme-linked immunosorbent assay was used to confirm HIV status*.* CD4 count was performed using the Becton Dickinson automated FACS count system [31]. Cervicitis was diagnosed from inflammatory cells by means of microscopic evaluation.

### Statistical analysis

Data were checked and cleaned as per standard processes without substantial implications to the data, and analysis was undertaken using STATA version 12 (Corporation, College Station, TX, USA).

We first described the distribution of pHR and HR HPV types observed among women with > CIN 2, CIN 2, CIN 3 and ICC.

Women over 30 years have a higher risk of abnormal cytology, hence, age was dichotomized into two categories, ≥30 years and < 30 years. CD4 cell count was transformed into categories including CD4 < 200 cells/μl and CD4 ≥ 200 cells/μl. This breakdown was used to assess severe immunosuppression. The number of pHR and HR HPV co-infections was treated both as a categorical variable, no HPV, one HPV and two or more HPV co-infections**.** As the outcome of interest, patients’ histology result was dichotomized into lower than CIN2 and CIN 2+. For the univariate analysis, a logistic regression was fitted to measure the strength of the association between pHR and HR HPV genotype separately and CIN 2+. A multivariable logistic regression analysis was performed to simultaneously control for potential confounders, including age, low CD4 count, and the presence of co-infections.

Based on women with CD4 count < 200 being more at risk for abnormal cytology, we tested this variable as a potential effect modifier. We fitted a regression model to assess the association between pHR and HR HPV genotypes on CIN2+; in the same model, we assessed the potential role of confounding and/or interaction of age and CD4 count. Statistical significance of an odds ratio (OR) was considered at *p* ≤ 0.05.

The log of the pHR/HR HPV viral load was taken as the data were not normally distributed.

## Results

### Characteristics of the population

This study consisted of 74 HIV+, non-pregnant women with an abnormal cytology, of which 81% (60/74) were on HAART. Our study population had a mean age of 34.2, and 73% of women were 30 years of age and older. The median CD4 count was 236 cells/μl [interquartile range (IQR) 158–374], and 35% had CD4 count of 200 cells/μl or lower. The median age at first sexual intercourse was 18 years (IQR = 15.5–20), and the median number of sex partners was 2 (IQR: 1–4).

### Prevalence of cervical and histological abnormalities

LSIL was detected in 43/74 (58%), Atypical Squamous cells of undetermined significance (ASC-US) in 12/74 (16%), Atypical Squamous Cells cannot rule Out High-Grade Squamous Intra-epithelial Lesion (ASC-H) in 3/74 (4%), (HSIL) in 15/74 (20%) and 1/74 was inconclusive (1%).

Histological results in the 74 women with cytological abnormalities were CIN 1 in 58% (41/74), CIN 2 in 16% (12/74), CIN 3 in 23% (17/74), and one participant (1/74) had invasive cervical carcinoma. Cervicitis was detected in 15% (11/74) and normal histology in 4% (3/74). Two (3%) biopsies were inconclusive. Overall, 30 (40.5%) women had a cytology of CIN2+ (CIN2, CIN3 and ICC combined).

### Prevalence of pHR and HR HPV genotypes

To assess the prevalence of, potential high risk (pHR) and high-risk (HR) HPV genotypes in the 74 HIV+ women with abnormal cytology, we assayed for specific genotypes listed in Table 1 by qPCR. In our study, 48 harbored (65%) at least one pHR/HR HPV genotype. The median number of concurrent HR HPV genotype infections was two (IQR: 2–4).

**Table 1:** Prevalence of pHR and HR HPV genotypes and pairs according to histological results

	Histological results			
HPV Genotype	< CIN 2 (*n* = 44)	CIN 2 (*n* = 12)	CIN 3 (*n* = 18)	ICC
HPV 16	32% (14/44)	25% (3/12)	44%(8/18)	
HPV 53	25% (11/44)	17% (2/12)	28% (5/18)	1
HPV 52	16% (7/44)	42% (5/12)	17% (3/18)	
HPV 56	18% (8/44)	8% (1/12)	33% (6/18)	
HPV 58	16% (7/44)	42% (5/12)	6% (1/18)	
HPV 18	16% (7/44)	25% (3/12)	17% (3/18)	
HPV 35	14% (6/44)	25% (3/12)	18% (4/18)	
HPV 31	7% (3/44)	33% (4/12)	18% (4/18)	
HPV 33	7% (4/44)	25% (3/12)	28% (5/18)	
HPV 39	7% (3/44)	17% (2/12)	17% (3/18)	
HPV 45	0%	8% (1/12)	11% (2/18)	
HPV 59	2% (1/44)	8% (1/12)	11% (2/18)	
HPV 66	20% (9/44)	8% (1/12)	9% (1/18)	
HPV 68	7% (3/44)	0% (0/12)	9% (1/18)	
Multiple HPV coinfection	60% (26/44)	83% (10/12)	67% (12/18)	

Among the 30 women with CIN 2+, over half of women (57%) had either HPV 16 or HPV 18 infection. The combined prevalence of intermediate HPV risk types in CIN 2+ was 30%, of which HPV 53 represented (7/30) 23% and HPV 66 (2/30) 7%. In women with CIN 3+, HPV 16 8/18 (44%), HPV 56 6/18 (33%), HPV 33 and 53 HPV 53 5/18 (28%) were the most prevalent genotypes (Table 1).

### HPV correlates of CIN 2+

Only one genotype, HPV 31, was found to be statistically significant in association with CIN 2+ adjusted for CD4 count, age and co-infections, (AOR = 4.9, 95%CI: 1.1–22.6). No interaction with CD4 count and age was noted (Table 2).

**Table 2:** Association between various pHR and HR HPV genotypes and CIN 2/+. Odds Ratios (OR) from logistic regression

Variables	adjusted OR for CIN 2+ (95%CI): model 1	*P*-value	adjusted OR for CIN 2+ (95%CI): model 2	*P*-value
Age 30 and above	0.9 (0.3–2.3)	0.8		
pHR and HR co-infections prevalence	1.6 (0.6–4.5)	0.4		
CD4 count < 200	2.2 (0.8–5.8)	0.2		
HPV 16	1.3 (0.5–3.6)	0.6	1.2 (0.4–3.5)	0.7
HPV 18	1.2 (0.3–4.2)	0.8	1.1 (0.3–3.9)	0.9
HPV 31	5.0 (1.1–21.6)	0.03	4.9 (1.03–22.5)	0.05
HPV 33	3.0 (0.8–11.5)	0.1	2.9 (0.7–11.1)	0.1
HPV 35	1.6 (0.5–4.4)	0.5	1.4 (0.4–5.0)	0.6
HPV 39	2.6 (0.6–12.3)	0.2	2.4 (0.5–11.6)	0.3
HPV 51	1.7 (0.4–6.4)	0.4	1.6 (0.4–6.1)	0.5
HPV 52	1.6 (0.5–5.1)	0.5	1.3 (0.4–4.7)	0.6
HPV 53	0.6 (0.2–2.0)	0.4	0.5 (0.1–1.7)	0.3
HPV 56	1.6 (0.5–5.6)	0.4	1.4 (0.4–5.2)	0.6
HPV 58	1.0 (0.3–4.0)	1.0	1.0 (0.3–3.5)	1.0
HPV 66	0.2 (0.04–1.0)	0.06	0.2 (0.03–0.9)	0.04
HPV 68	0.5 (0.05–5.4)	0.4	0.2 (0.04–4.5)	0.5

Model 1: adjusted for CD4 count, age

Model 2: adjusted for CD4 count, age, and pHR and HR HPV coinfections

P-value from Likelihood Ratio Test

We found a non-significant inverse association between HPV 53 and CIN 2+, and a significant inverse effect against HPV 66 when adjusted for co-infection (Table 3). Seventy-three percent (22/30) of women with CIN 2+ harbored 2 or more pHR and HR HPV genotypes. A univariate logistic regression yielded a statistically non-significant association (OR: 1.9; *p* = 0.2; CI: 0.7–5.3) between CIN 2+ and multiple pHR and HR HPV genotypes (Table 3).

**Table 3:** Mean log viral load copies/ 10^3^ cells per pHR and HR HPV genotypes

pHR and HR HPV genotypes	Mean VL copies in CIN 1 (95% CI)	Mean VL copies in CIN 2+ (95% CI)	*P* value
HPV 16	8.5(6.6–10.4)	11.2 (9.0–13.4)	0.05
HPV 18	7.1 (4.4–9.8)	6.4 (2.8–9.9)	0.7
HPV 31	6.2 (0–14.8)	10.4 (7.0–13.8)	0.1
HPV 33	9.5 (6.3–12.7)	11.4 (9.8–13.1)	0.1
HPV 35	11.1 (6.1–16.0)	8.5 (6.4–10.5)	0.2
HPV 39	9.2 (0.1–18.2)	7.8 (5.3–10.3)	0.5
HPV 45			
HPV 51	9.8 (3.5–16.1)	9.3 (5.9–12.6)	0.8
HPV 53	6.0 (3.9–8.2)	3.4 (1.3–5.4)	0.07
HPV 56	11.0 (8.7–13.3)	9.1 (5.8–12.5)	0.3
HPV 58	8.1 (6.1–10.0)	8.3 (4.3–12.1)	0.9
HPV 66	6.4 (0–19.4)	8.9 (6.6–11.2)	0.3
HPV 68	6.0 (0–15)	NA	NA
Total pHR/HR HPV VL	10.7 (9.6–11.8)	11.8 (10.7–12.9)	0.2

To assess the specific pHR/HR HPV load as a type-dependent risk marker for CIN 2+, we measured the log viral load copies/10^3^ cells for specific genotypes in the 74 HIV+ women. Among the 30 women with CIN 2+, HPV 16 and its phylogenetically related HPV 31 and 33 were found to have the highest mean viral load, with HPV 16 having a mean of 11.2 (9.0–13.4) (Table 3).

## Discussion

We observed a combined HPV 16 and HPV 18 prevalence of 57% in women with CIN 2+ and a prevalence of pHR HPV genotypes of 30%. In line with recent findings of a meta-analysis on HPV distribution in HIV-infected women disaggregated by cytological status in Africa, we found a higher prevalence of HPV 16 in women with CIN 3 (56%) than CIN 2 (25%), although our percentage for CIN 3 was higher than the one reported (41%–47%) [32].

Contrary to our first hypothesis, our data do not suggest a significant association between multiple pHR/HR HPV infections and CIN 2+. This study suggests that HPV 31 is the only independent predictor of CIN 2+, and an inverse relationship was detected between HPV 66 and CIN 2+. However, in agreement with our hypothesis, our study suggests that HPV 16 viral load correlated with CIN 2+. Our combined HPV 16 and HPV 18 prevalence of 61% in CIN 3+ suggests the need for a wider protection that the nonavalent vaccine would confer.

There is a presumed link between HIV-positivity and the prevalence of multiple HPV infections [10, 11]. Our non-significant association between 2 or more coinfections and CIN 2+ contrasts with findings of a recent large study on multiple HPV infections in Costa Rica in which 5871 young healthy women with multiple infections were at significantly increased risk of CIN 2+ when compared with those with single infections [33].

Contrary to our hypothesis of required synergistic mechanisms between pHR/HR HPV genotypes for cervical cancer genesis, it may be that in our study population with a low median CD4 count of 236 cells/μl (IQR: 158–374), single pHR HPV genotypes are capable of inducing cervical cancer genesis.

Our lack of association between pHR HPV genotypes and CIN 2+ is in agreement with an observation made by Rahman et al. (2011) that infection with pHR HPV genotypes in HSIL became non-significant when HIV status was included in the multivariate analysis. In women with CIN 3+, HPV 53 was the third most prevalent genotype. (28%) The stand-alone HPV 53 in the only ICC case recorded is incongruent with those from a recent study in Kenya, where pHR HPV genotypes were only detected in low-grade lesion [34]. The woman with ICC was severely immunocompromised and had a very low CD4 count of 2 cells/μl, which we hypothesize may make her more at risk for a potential oncogenic capacity of a pHR HPV genotype.

When examining associations between specific pHR and HR HPV genotypes and CIN 2+, a multivariate analysis suggested the presence of HPV 31 as an independent predictor of CIN 2+. Furthermore, a high number of concomitant pHR and HR HPV infections in women was observed in the presence of HPV 31.

Our finding suggesting that HPV 16 viral load may correlate with the severity of lesions is congruent with those found in the literature pertaining to sub Saharan Africa [35, 36]. Notwithstanding, a high viral mean load found for HPV 31 and 33 in women with CIN 2+, a statistically significant association was not demonstrated for these HR HPV genotypes. From an epidemiological perspective, this finding suggests a higher replicative capacity for HPV 16, which is known to be persistent, along with its phylogenetically related genotypes HPV 31 and 33 in HIV-infected women with CIN 2+ and may result in an increased transmission rate. As a corollary, this underscores the public health impact of monitoring unvaccinated HIV-infected women more regularly than once every three years as recommended by the WHO [37].

Our findings can be extrapolated to a HIV population that is moderately to severely immunosuppressed and has had few sexual partners within the region. The relatively high median of concurrent pHR and HR HPV infections suggests an inability to either clear HPV infectious or a propensity to reactivate latent HPV infections. However, it is unknown how many sexual partners their spouses have had, nor can a social desirability bias in reporting sexual behavior be excluded.

### Strength and limitations

A major strength of our study is that our samples have been histopathologically confirmed. A recent systematic review suggests that most studies on cervical dysplasia on the continent, have cervical cytology as endpoints, [38] which only has a clinical sensitivity between 55%–65% for detection of histopathologically confirmed ‘true disease status [39]. Furthermore, the real-time TaqMan PCR assays we used for detecting HPV genotypes were validated [40].

We recognize that our study has certain inherent methodological limitations. The small sample size compromised our power to assess correlates solely for CIN 3, which is the best proxy for ICC, despite type distribution in CIN 3 not being completely representative of cancer [32].

Additionally, the cross-sectional design, which does not allow the fulfillment of the temporal criterion for causality, bases its analysis on a single measure of pHR and HR HPV viral load for prevalent infections at the baseline screening phase, which may not be able to capture the transient nature of pHR and HR HPV infections. Consequently, it may not be possible to disentangle the risk posed by recently acquired infections along with its elevated viral load from viral load deriving from older infections.

A further limitation related to a cross sectional study design may be the lack of data concerning age of acquisition of HIV infection, since it is possible this may have occurred too late in life for some of the women in our study to influence CIN 2+. Moreover, lack of data on HIV viral load and on the recombinant strains present in HIV-1 infected women, precludes us from fully exploring synergistic mechanisms between the two viruses.

### Research gaps

The epidemiology of pHR HPV genotypes in HIV women is still poorly characterized, as HPV 26, 53, 67, 70,73, and 82 are not included in any HPV DNA screening protocols in sub Saharan Africa. Our findings warrant that the potential carcinogenesis of HPV 53 be better elucidated, especially in severely immunosuppressed women. According to Padalko et al. (2015) the role of pHR HPV genotypes will also need to be assessed in the post vaccine era, in case type replacement leads to pHR HPV genotypes becoming more prevalent in ICC [41].

A systematic review and meta-analysis [42] found that the bivalent vaccine from GlaxoSmithKline had better cross protection against HPV 31 in persistent infection, but that efficacy against persistent infections with type 31 appeared to decrease with longer follow-up, suggesting a waning of cross-protection. It still remains to be determined whether a cross protection can be extrapolated to HIV-infected women and in the presence of multiple HR HPV genotypes.

The kinetics of different genotype viral load must also be assessed amidst, not only different levels of immunosuppression, but also in the presence of different levels of HIV viral load, multiple pHR and HR HPV infections and other concomitant sexually transmitted infections harbored by HIV-infected women. This would help to elucidate the aetiologic role of pHR and HR HPV viral load in cervical dysplasia pathogenesis and determine virological parameters to predict high-grade lesion in HIV infected women in sub Saharan Africa.

## Conclusion

Our small sample suggest a high prevalence of HPV 16, 53, 56 and 33 in women with CIN 3+. Furthermore, a HPV 31 was found to be an independent predictor of CIN 2+ and HPV 16 viral load significantly higher in women with CIN 2/+.

Whether the available bivalent prophylactic vaccine will be able to meet its objective of reducing cervical cancer incidence by 70% may depend on the efficacy of cross protection against HPV 31 in HIV-infected women and the synergies between HPV genotypes in inducing cervical cancer genesis. The high prevalence of non-HPV 16 and 18 genotypes underscore the benefits of the nonavalent vaccine within this population.

Our high prevalence of HPV 53 in CIN 3+ and as a stand-alone HPV genotype in ICC, suggests a need for enhanced HPV 53 detection and its incorporation into a local screening protocol. Given the potential public health impact of pHR HPV in HIV-infected women and its exclusion from prophylactic vaccines, future research efforts are needed to investigate the epidemiology of these genotypes in HIV-infected women in the role of cervical cancer genesis. Moreover, large protective studies assessing the impact of the kinetics on different genotype viral load in HIV infected women in sub Saharan Africa, are needed to elucidate the tripartite relationship between HIV viral load, CD4 count and pHR and HR HPV viral load.

## Abbreviations

ASC-H: Typical squamous cells-cannot exclude high-grade squamous intraepithelial lesion
ASC-U: Atypical cells of undetermined significance
CIN: Cervical intraepithelial neoplasia
HAART: Highly active antiretroviral therapy
HIV: Human immunodeficiency virus
HPV: Human Papilloma virus
HSIL: High grade squamous intraepithelial lesion
ICC: Invasive Cervical Cancer
LEEP: loop electrosurgical excision procedure (LEEP) ECC endocervical curettage
LSIL: Low grade squamous intraepithelial lesion.
pHR HPV: potential high risk
